# Aprepitant, an antiemetic agent, interferes with metal ion homeostasis of *Candida auris* and displays potent synergistic interactions with azole drugs

**DOI:** 10.1080/21505594.2020.1838741

**Published:** 2020-10-26

**Authors:** Hassan E. Eldesouky, Nadia A. Lanman, Tony R. Hazbun, Mohamed N. Seleem

**Affiliations:** a Department of Comparative Pathobiology, College of Veterinary Medicine, Purdue University, West Lafayette, IN, USA; b Department of Biomedical Sciences and Pathobiology, Virginia-Maryland College of Veterinary Medicine, Virginia Polytechnic Institute and State University, Blacksburg, VA, USA; c Purdue University Center for Cancer Research, Purdue University, West Lafayette, IN, USA; d Department of Medicinal Chemistry and Molecular Pharmacology, College of Pharmacy, Purdue University, West Lafayette, IN, USA

**Keywords:** *Candida auris*, fungal biofilms, azole resistance, *Caenorhabditis elegans*, metal ion homeostasis, reactive oxygen species (ROS)

## Abstract

With the rapid increase in the frequency of azole-resistant species, combination therapy appears to be a promising tool to augment the antifungal activity of azole drugs against resistant *Candida* species. Here, we report the effect of aprepitant, an antiemetic agent, on the antifungal activities of azole drugs against the multidrug-resistant *Candida auris*. Aprepitant reduced the minimum inhibitory concentration (MIC) of itraconazole *in vitro*, by up to eight-folds. Additionally, the aprepitant/itraconazole combination interfered significantly with the biofilm-forming ability of *C. auris* by 95 ± 0.13%, and significantly disrupted mature biofilms by 52 ± 0.83%, relative to the untreated control. In a *Caenorhabditis elegans* infection model, the aprepitant/itraconazole combination significantly prolonged the survival of infected nematodes by ~90% (five days post-infection) and reduced the fungal burden by ~92% relative to the untreated control. Further, this novel drug combination displayed broad-spectrum synergistic interactions against other medically important *Candida* species such as *C. albicans, C. krusei, C. tropicalis*, and *C. parapsilosis* (ƩFICI ranged from 0.08 to 0.31). Comparative transcriptomic profiling and mechanistic studies indicated aprepitant/itraconazole interferes significantly with metal ion homeostasis and compromises the ROS detoxification ability of *C. auris*. This study presents aprepitant as a novel, potent, and broad-spectrum azole chemosensitizing agent that warrants further investigation.

## Introduction

*Candida auris* is an emerging fungal pathogen that has been recently implicated in numerous global outbreaks of life-threatening candidemia, leading to significant morbidity and high rates of mortality [1,2]. Recently, the U.S. Centers for Disease Control and Prevention (CDC) has classified *C. auris* as an urgent threat that requires immediate intervention [3]. Unfortunately, resistance to major antifungal agents, including to the first-line therapeutics, was reported to be common among *C. auris* isolates [4]. According to the CDC, 90% of *C. auris* isolates are resistant to the antifungal activity of fluconazole and around 30% of isolates are resistant to amphotericin B [5]. The unique multidrug resistance nature of *C. auris* combined with its ability to be efficiently transmitted among patients are key challenges that hinder the development of effective control and containment of *C. auris* [1,6–8].

Currently, only three classes of systemic antifungal drugs (azoles, polyenes, and echinocandins) are available for the treatment of invasive *Candida* infections [9]. Azole drugs target ergosterol biosynthesis through the inhibition of lanosterol 14 alpha methyltransferase (Erg11p) resulting in depletion of the vital cell membrane component, ergosterol, and accumulation of cytotoxic metabolites [10]. Owing to safety, the spectrum of activity, and pharmacokinetic considerations such as oral bioavailability, azole antifungal drugs have gained preference as vital therapeutics to control various types of mycotic infections [11–14]. However, the extensive dependence on azole drugs has contributed to the increased rate of azole-resistance among fungal species [15,16]. Overexpression or mutation of *ERG11*, in addition to the upregulation of ABC and MFS efflux transporters, are known to be major triggers of azole resistance in *Candida* species [17].

Considering the limited therapeutic options and the increased rate of drug resistance in *C. auris*, there is a pressing need for novel antifungal agents and co-drugs capable of restoring or enhancing the antifungal activity of azole drugs. Historically, drug combinations have been utilized efficiently to treat various bacterial and viral infections and to fight cancer [18–21]. However, this approach has not been fully exploited in medical mycology despite the immediate need to discover new treatment strategies, especially with the current dearth in available therapeutics and the increased frequency of multidrug-resistant isolates.

In a whole-cell screening study aimed at identifying novel azole chemosensitizing agents (unpublished data), we noticed that the antiemetic agent aprepitant was able to restore the antifungal activity of fluconazole against the test *C. auris* isolate. Here, we investigated the interactions between aprepitant and different azole drugs against multiple *C. auris* isolates and other clinically important *Candida* species. Additionally, comparative transcriptomic profiling and mechanistic studies were performed to identify the potential mechanism by which aprepitant enhances the antifungal activities of azole drugs.

## Materials and methods

### Fungal strains, reagents, and chemicals

Sources and descriptions of the fungal strains used in this study are provided in Supplementary Table S1. Yeast-peptone dextrose (YPD) agar and broth were purchased from Becton, and Dickinson Company (Franklin Lakes, NJ). 3-(N-Morpholino) propanesulfonic acid (MOPS) was purchased from Sigma-Aldrich (St. Louis, MO). RPMI 1640 powder, supplemented with glutamine and lacking NaHCO_3_, was obtained from Thermo Fisher Scientific (Waltham, MA). Johns Hopkins Clinical Compounds Library (JHCCL) was obtained from the school of medicine at Johns Hopkins University and was used to identify the azole chemosensitizing activity of aprepitant. The library was screened at 16 µM against *C. auris* AR0390 in the presence or absence of a subinhibitory concentration of fluconazole (32 µg/ml). Fluconazole was purchased from Fisher Scientific (Pittsburgh, PA). Itraconazole, voriconazole, and aprepitant were purchased from TCI America (Portland, OR). Gentamicin sulfate was obtained from Chem-Impex International Inc. (Wood Dale, IL).

### Microdilution checkerboard assays

Aprepitant and azole drugs (fluconazole, voriconazole, and itraconazole) were tested alone and in combinations against different *Candida* species using standard broth microdilution checkerboard assays, as previously described [22–24]. The ΣFICI (fractional inhibitory concentration index) was used to evaluate the interactions between the tested drugs. Interactions were deemed to be synergistic (SYN) when ΣFICI values were ≤0.50, additive (ADD) when ΣFICI values were >0.50 and ≤1, and indifferent (IND) when ΣFICI values were >1 and ≤4 [25].

### Time-kill assay

To study the effect of aprepitant/itraconazole on the growth kinetics of *C. auris*, a time-kill assay was conducted, as previously described [26–29]. Briefly, exponential-phase *C. auris* AR0390 cells were diluted with RPMI 1640 medium to ~1 × 10^5^ CFU/ml. Cells were incubated with aprepitant (10 µg/ml), itraconazole (1 µg/ml), or a combination of both agents for 24 h at 35°C. Following incubation, the number of viable cells was determined at specific time points (0, 6, 12, and 24 h). The time-kill curve was constructed by plotting viable colony forming units against time. The results are presented as the average values of triplicate measurements.

### Effect of aprepitant/itraconazole combination against premature and mature C. auris biofilms

To assess the antibiofilm activity of aprepitant/itraconazole against *C. auris*, overnight cultures of *C. auris* AR0390 (~1 × 10^5^ CFU/ml) in RPMI 1640 were transferred onto tissue culture-treated 96-well plates and incubated for 2 h at 35°C to allow cell adhesion. Then aprepitant (10 µg/ml), itraconazole (1 µg/ml), or a combination of both agents were added, and the plates were incubated for 24 h at 35°C. Cells treated with DMSO (1%), the drugs’ solvent, served as a negative control. Following incubation, RPMI 1640 medium was discarded and the formed biofilms were carefully washed twice with PBS before being quantified, using the XTT reduction assay [26,30].

To assess the activity of aprepitant/itraconazole against mature biofilms, *C. auris* cells (at ~1 × 10^5^ CFU/ml) were seeded in tissue culture-treated 96-well plates and incubated for 24 h at 35°C to allow for the formation of mature biofilms [31–33]. Fresh RPMI 1640 media supplemented with aprepitant, itraconazole, or aprepitant/itraconazole, at the same concentrations indicated above, were added to the formed biofilms and incubated for another 24 h at 35°C. After incubation, biofilms were carefully washed and quantified by the XTT reduction method. All treatments were conducted in triplicates and data represent the average ± SD.

### Effect of aprepitant/itraconazole against other Candida species

To investigate the effect of aprepitant on the antifungal activity of itraconazole against other medically important *Candida* species, checkerboard assays were utilized and ΣFIC indices were calculated as mentioned above. The aprepitant/itraconazole combination was assessed against *C. albicans* (n = 4), *C. glabrata* (n = 3), *C. krusei* (n = 3), *C. tropicalis* (n = 2), and *C. parapsilosis* (n = 2). Additionally, 5 µl samples from representative *Candida* cultures that displayed enhanced susceptibility to the aprepitant/itraconazole combination were spotted onto YPD agar plates and incubated for 24 h at 35°C before scanning the plates and determination of CFU counts.

### RNA extraction

Exponential-phase cultures of *C. auris* AR0390 were treated (in duplicates) with DMSO (1%), aprepitant (10 µg/ml), itraconazole (1 µg/ml), or a combination of both drugs. All treated cultures were incubated for 3 h at 30°C. The cells were pelleted, washed twice with PBS, and RNA was isolated using an Ambion Ribopure yeast kit. The RNA quality was checked on a bioanalyzer Nano chip (Agilent) and all RNAs used for downstream experiments were determined to have RNA integrity numbers (RIN) of 9.5 and above. The SuperScript III First-Strand kit (Invitrogen) was used to prepare cDNA, following the manufacturer’s guidelines.

### RNA sequencing and enrichment analysis of differentially expressed genes (DEGs)

Paired-end, 2 × 150 bp reads were sequenced on a NovaSeq6000. Reads were quality trimmed and Illumina TruSeq adapter sequences were removed using fastp [34]. HISAT2 (v2.1) was used to align reads to the *Candida auris* NCBI reference genome version B11221 [35]. FeatureCounts (v2.1) was used to count the read numbers mapped of each gene [36]. FPKM values of each gene were calculated based on the length of the gene in the associated annotation GTF file as well as reads count mapped to each gene. Differential expression analysis was performed on raw count data using the edgeR Bioconductor package (v3.16.5) [37,38]. The *P* values were adjusted using the Benjamini & Hochberg method [39]. A corrected *P*-value of 0.005 and an absolute foldchange of two was set as the threshold for significant differential expression. Differentially expressed genes were annotated by blasting the sequences of identified DEGs against the *Candida* Genome Database (CGD) and by using blasts against *C. albicans* SC5314 (taxid:237561) with an e-value cutoff of 0.01 [40]. Gene Ontology (GO) enrichment analysis of differentially expressed genes was implemented by the ClusterProfiler Bioconductor package (v2.4.3) [41]. GO terms with corrected *P*-values < 0.05 were considered significantly enriched within the differentially expressed genes lists.

### Real-time quantitative reverse transcription (RT-qPCR)

To validate the RNA-Seq results, we conducted quantitative real-time (RT) PCR assays to quantify the expression of six genes (*ERG1, ERG2, ERG10, FRT1, ZRT2*, and *CTR1*). Gene expression levels were calculated using the 2^−ΔΔCT^ method [42]. Gene expression was internally normalized to *ACT1* and compared to the untreated control (DMSO 1%). All primers used in this study were provided in Supplementary Table S2.

### Effect on ROS levels in C. auris cells

The Image-iT™LIVE Green Reactive Oxygen Species (ROS) detection kit (Molecular Probes, Inc., Eugene, OR) was utilized to measure ROS levels, as previously described [27]. Briefly, exponential-phase *C. auris* AR0390 was adjusted to ~ 1 × 10^6^ CFU/ml in PBS in the presence or absence of FeSO4 (50 µM). Cells were treated with either aprepitant (10 µg/ml), itraconazole (1 µg/ml), or a combination of both drugs, while DMSO (1%) treated culture was used as a negative control. All treatments were continued for 3 h at 35°C, then cells were pelleted, washed twice with PBS, and 5-(and-6)-carboxy-2′,7′-dichlorodihydrofluorescein diacetate (carboxy-H2DCFDA) dye, at a final concentration of 50 µM, was added. Cells were then incubated for 1 h at 35°C. After incubation, cells were washed twice with PBS and the fluorescence intensity was measured spectrophotometrically at 495/529 nm. Data represent the average of three measurements ±SD.

### Effect of iron supplementation on the antifungal activity of aprepitant/itraconazole

To investigate the effect of iron supplementation on the synergistic relationship between aprepitant and itraconazole against *C. auris*, a time-kill assay was performed as described above, and RPMI 1640 with/without ferrous sulfate (50 µM) was used as the assay medium. Cultures were incubated at 35°C for 24 h and then 5 µl samples of each treatment were spotted onto YPD agar plates and incubated at 35°C for another 24 h before scanning the plates.

### Caenorhabditis elegans infection model

To assess the *in vivo* efficacy of the aprepitant/itraconazole combination, a *C. elegans* infection model was utilized as previously discribed [9,11,26]. Briefly, synchronized worms [strain AU37 genotype glp-4(bn2) I; sek-1(km4) X] at L4 phase were incubated in 5 ml of YPD broth containing *C. auris* AR0390 (adjusted at ~1 × 10^6^ CFU/ml) for 3 h at room temperature. Following infection, worms were washed five times with PBS to remove non-ingested cells and then suspended in M9 buffer containing 20% RPMI 1640. Infected worms were distributed into groups (~20 worms/group) and then treated (in triplicates) with either DMSO (1%), aprepitant (10 µg/ml), itraconazole (1 µg/ml), or a combination of both drugs. Worms were then incubated with test agents at 25°C for 24 h before being washed twice with PBS and beaten vigorously with silicon carbide beads, for at least 2 min, to release *C. auris* cells. *C. elegans* lysates were serially diluted and plated over YPD agar plates containing gentamicin (100 µg/ml). Plates were incubated for 24 h at 35°C before viable CFU per worm was determined.

In another experiment, worms were incubated in 5 ml of YPD broth containing *C. auris* AR0390 (adjusted at ~1 × 10^6^ CFU/ml) for 3 h at room temperature. Infected worms were treated with DMSO (1%), aprepitant (10 µg/ml), itraconazole (1 µg/ml), or a combination of both drugs. The survival of *C. elegans* was monitored and recorded for five days. Data are presented as percent survival of infected *C. elegans* using a Kaplan-Meier survival curve generated using GraphPad Prism 6.0 (GraphPad Software, La Jolla, CA).

### Statistical analyses

Statistical analysis was performed using GraphPad Prism 6.0 (Graph Pad Software, La Jolla, CA, USA). One-way ANOVA and Dunnett’s test for multiple comparisons were used to assess the statistical significance (*P* < 0.05) between treated and untreated groups. Kaplan-Meier survival curves were assessed by the log-rank test for statistical significance.

## Results

### Azole chemosensitizing activity of aprepitant against C. auris isolates

To identify novel adjuvants to overcome azole resistance in *C. auris*, we explored the azole chemosensitizing activity of ~1600 FDA-approved drugs in the Johns Hopkins Chemical Library (unpublished data). Our initial screen identified aprepitant (an antiemetic agent) as a potent hit compound that restored the antifungal activity of fluconazole against the multidrug-resistant strain *C. albicans* AR0390. This observation encouraged us to explore the interactions between aprepitant and commonly used azole drugs *in vitro* against a panel of ten *C. auris* isolates obtained from the Antimicrobial Resistance Isolate Bank (CDC). To assess these interactions, we used standard microdilution checkerboard assays and calculated the fractional inhibitory concentration indices (ƩFICI). As shown in Table 1, aprepitant was able to enhance the antifungal activity of fluconazole against seven isolates with a synergistic relationship observed against four isolates (ƩFICI ranged from 0.09 to 0.50) and an additive relationship observed against three isolates (ƩFICI ranged from 0.51 to 0.63). Based on the current tentative breakpoints for azole resistance, aprepitant was able to restore the fluconazole susceptibility only in two fluconazole-resistant isolates, AR0388, and AR0390. When tested in combination with voriconazole, aprepitant was able to enhance the antifungal activity of voriconazole against eight isolates, with a synergistic relationship observed against two isolates (ƩFICI = 0.19) and an additive relationship observed against six isolates (ƩFICI ranged from 0.53 to 0.56). However, aprepitant failed to re-sensitize any of the tested voriconazole-resistant isolates (n = 3) that exhibited MIC ≥1 µg/ml. Interestingly, when combined with itraconazole, aprepitant was able to enhance the antifungal activity of itraconazole against all ten tested isolates, resulting in a synergistic relationship observed against eight isolates (ƩFICI ranged from 0.14 to 0.31) and an additive relationship observed against two isolates (ƩFICI = 0.52). Importantly, aprepitant was able to re-sensitize all tested isolates (n = 3) that displayed reduced susceptibility to itraconazole (MIC ≥1 µg/ml). Since aprepitant interacted more favorably with itraconazole against *C. auris*, the combination between aprepitant and itraconazole was selected for further investigation.

**Table 1.** Effect of aprepitant on the antifungal activity of fluconazole (FLC), voriconazole (VRC), and itraconazole (ITC) against *C. auris* clinical isolates

*C. auris* Isolates	MIC (µg/ml)				Combination with FLC			Combination with VRC			Combination with ITC		
	APR	FLC	VRC	ITC	APR/FLC (µg/ml)	ƩFIC	Mode	APR/VRC (µg/ml)	ƩFIC	Mode	APR/ITC (µg/ml)	ƩFIC	Mode
AR0381	> 128	1	0.0078	0.25	0.5/0.5	0.50	SYN	8/0.007	1.06	IND	2/0.125	0.52	ADD
AR0382		1	0.0625	0.25	1/0.5	0.51	ADD	8/0.031	0.56	ADD	8/0.0312	0.19	SYN
AR0383		256	0.5	0.5	2/256	1.02	IND	8/0.25	0.56	ADD	8/0.125	0.31	SYN
AR0384		128	0.5	0.25	2/64	0.52	ADD	8/0.25	0.56	ADD	8/0.0312	0.19	SYN
AR0385		256	4	0.5	2/256	1.02	IND	4/2	0.53	ADD	8/0.125	0.31	SYN
AR0386		256	2	0.5	16/128	0.63	ADD	4/1	0.53	ADD	8/0.125	0.31	SYN
AR0387		1	0.0312	0.125	0.5/0.5	0.50	SYN	4/0.015	0.53	ADD	8/0.0312	0.31	SYN
AR0388		256	0.5	1	8/8	0.09	SYN	8/0.062	0.19	SYN	2/0.125	0.14	SYN
AR0389		256	2	1	8/256	1.06	IND	8/2	1.06	IND	2/0.5	0.52	ADD
AR0390		256	0.5	1	8/8	0.09	SYN	8/0.062	0.19	SYN	4/0.125	0.16	SYN

*ΣFICI (fractional inhibitory concentration index) was used to measure the interaction between the tested combinations. ΣFICI interpretation corresponded to the following definitions: synergism (SYN), ΣFICI ≤0.5; additivity (ADD), ΣFICI >0.5 and ≤1; and indifference (IND), ΣFICI >1 and ≤4.

### Aprepitant/itraconazole exerts a fungicidal effect against C. auris AR0390

**Figure 1.**
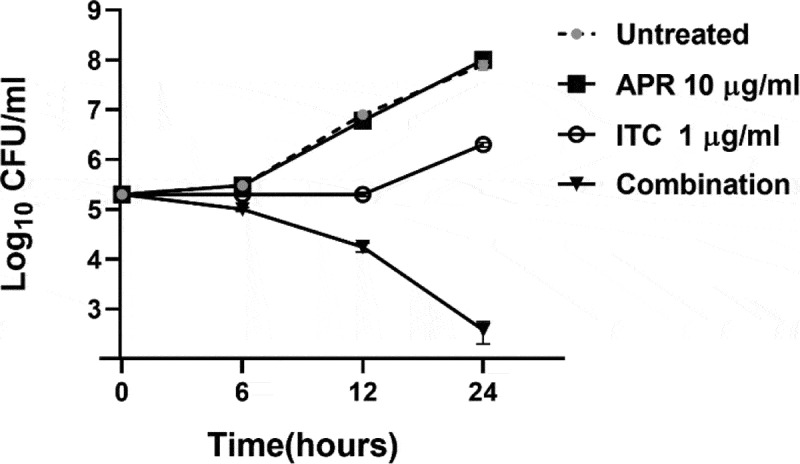
Time-kill assay of aprepitant (APR) at 10 µg/ml, itraconazole (ITC) at 1 µg/ml, or a combination of both agents at the same concentration as tested alone. Test agents were evaluated against *C. auris* AR0390 over a 48-hour incubation period at 35 °C. Cells treated with DMSO (1%) served as a negative untreated control. Error bars represent standard deviation values

To investigate the killing kinetics of the aprepitant/itraconazole combination against *C. auris*, a time-kill assay was conducted. As shown in Figure 1, aprepitant (at 10 µg/ml), by itself, did not produce any observable antifungal activity against the test isolate (AR0390). Itraconazole, at 1 × MIC (1 µg/ml), exhibited fungistatic activity for 12 h post-incubation; however, rapid fungal re-growth was observed thereafter. Interestingly, the combination of aprepitant and itraconazole (at the same concentrations each drug was tested alone) displayed fungicidal activity against the test isolate generating ~ 3-log_10_ reduction in the CFU count over 24 hours.

### Effect of aprepitant/itraconazole against C. auris biofilms

**Figure 2.**
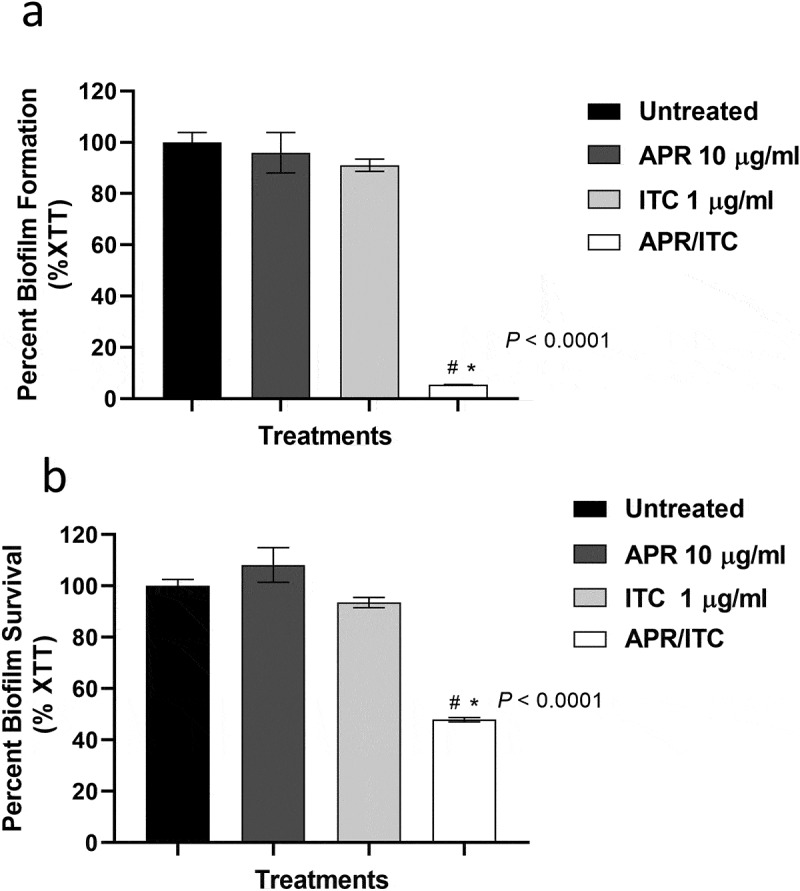
The anti-biofilm activity of aprepitant (APR)/itraconazole (ITC) against *C. auris*. (a) The inhibitory effect of the aprepitant (10 µg/mL)/itraconazole (1 µg/mL) combination on the formation of *C. auris* AR0390 biofilms as determined by the XTT reduction assay. (b) The ability of the aprepitant/itraconazole combination to diminish the metabolic activity of mature *C. auris* AR0390 biofilms. Data are shown as means ± SD. * indicates a statistical significance (*P* < 0.05) relative to the untreated control (*P* < 0.05), whereas #, indicates a statistical significance (*P* < 0.05) relative to the treatment with either aprepitant or itraconazole alone. Statistical significance was assessed by one-way ANOVA using Dunnett’s test for multiple comparisons

As shown above, the aprepitant/itraconazole combination exerted fungicidal activity against planktonic *C. auris*. To investigate whether a similar effect could be observed against *C. auris* biofilms, we utilized the XTT reduction assay to examine the effect of aprepitant/itraconazole against early-stage and mature biofilms. As shown in Figure 2(a), the combination of aprepitant (at 10 µg/ml) and itraconazole (at 1 µg/ml) significantly inhibited the biofilm-forming ability of the test isolate by ~95 ± 0.13% compared to the untreated control. However, single treatments with either aprepitant or itraconazole at the respective concentrations failed to interfere with the biofilm-forming ability of the test isolate.

When tested against mature *C. auris* biofilms, the aprepitant/itraconazole combination, at the same concentrations, was able to significantly reduce the viability of *C. auris* AR0390 cells present in mature biofilms by 52 ± 0.83% (Figure 2(b)). As expected, neither aprepitant nor itraconazole alone, at the respective concentrations, was able to interfere with the viability of *C. auris* cells within mature biofilms. These data indicate the potent ability of aprepitant/itraconazole to interfere with both the early and late phases of *C. auris* biofilms.

### Effect of aprepitant/itraconazole against other Candida species

**Figure 3.**
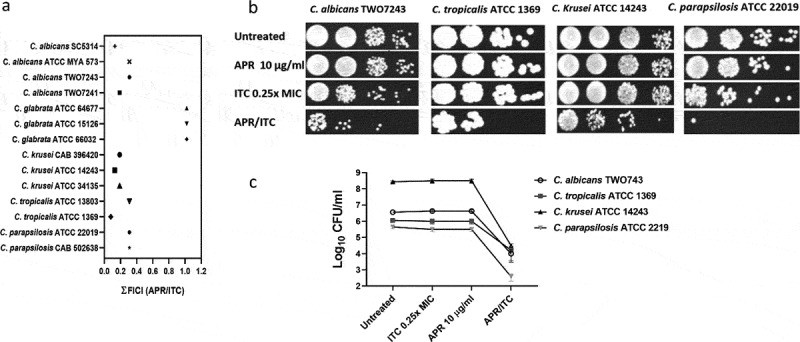
Interaction between aprepitant and itraconazole against different *Candida* species. (a) ƩFICI (fractional inhibitory concentration index) values were calculated from checkerboard assays. Cultures of *C. albicans* TWO7243, *C. krusei* ATCC 14243, *C. tropicalis* ATCC 1369, and *C. parapsilosis* ATCC 22019 were treated with APR (10 µg/ml) and ITC (0.25 × MIC), either alone or in combination. Treated cultures were incubated at 35°C for 24 h before being spotted onto YPD agar plates and reincubated for an additional 24 h. Plates were then scanned (b) and CFU count was performed (c)

The potent synergistic interaction observed between aprepitant and itraconazole against all *C. auris* isolates tested encouraged us to examine whether the aprepitant/itraconazole combination would exert a similar effect against other clinically important *Candida* species. Using standard microdilution checkerboard assays, we examined the interactions between aprepitant and itraconazole against a panel of 14 *Candida* species including *C. albicans* (n = 4), *C. glabrata* (n = 3), *C. krusei* (n = 3), *C. tropicalis* (n = 2), and *C. parapsilosis* (n = 2). As shown in Figure 3(a) and Supplementary Table S3, aprepitant exerted a synergistic relationship with itraconazole (ƩFICI ranged from 0.08 to 0.31) against all isolates, except for *C. glabrata* isolates where an indifferent effect was observed (ƩFICI = 1.02). These synergistic interactions were further demonstrated using spot assays. As shown in Figure 3(b), the aprepitant/itraconazole combination inhibited the visual growth of *C. albicans* (TWO7243), *C. krusei* (ATCC 14243), *C. tropicalis* (ATCC 1369), and *C. parapsilosis* (ATCC 22019), and reduced the CFU counts by ~ 2–4 orders of magnitude relative to the untreated control, as shown in Figure 3(c).

### Transcriptomic profiling of C. auris treated with aprepitant/itraconazole

**Figure 4.**
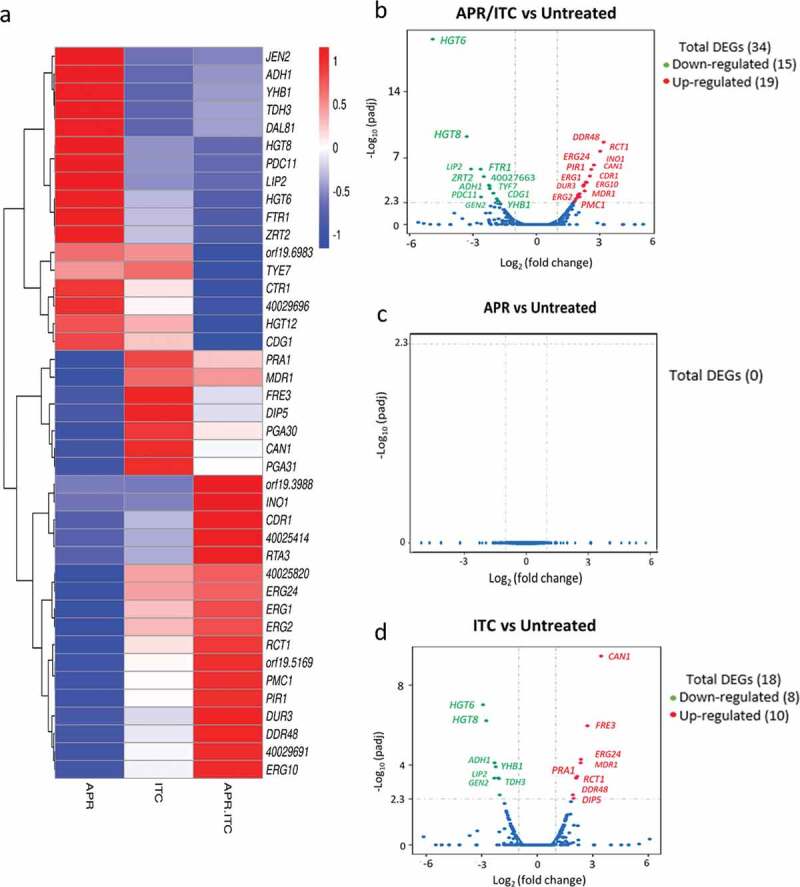
Transcriptional comparison of *C. auris* AR0390 treated with aprepitant (APR), itraconazole (ITC), or a combination of both versus the untreated control. (a) Heat map of FPKM values of DEGs of each treatment versus the untreated control, scaled by row. (b) Volcano blot of DEGs from *C. auris* AR0390 treated with aprepitant/itraconazole (APR/ITC) at 10/1 µg/ml. (c) Volcano blot of DEGs from *C. auris* AR0390 treated with aprepitant (APR) at 10 µg/ml. (d) Volcano blot of DEGs from *C. auris* AR0390 treated with itraconazole (ITC) at 1 µg/ml. Significant down-regulated DEGs are shown in green, whereas significant up-regulated DEGs are shown in red

To gain insight into the molecular mechanism(s) by which aprepitant interacts synergistically with azole drugs, we performed comparative transcriptomic analysis of *C. auris* AR0390 treated with DMSO (1%), aprepitant (10 µg/ml), itraconazole (1 µg/ml), or a combination of both drugs. A total of 81.9 million reads were obtained with over 95% mapping rates observed across all samples. The sequence reads of all samples were deposited in the NCBI sequence read archive (GEO) under the accession number of (GSE148749). The Bioconductor package edgeR was used to identify differentially expressed genes (DEGs) between groups. Genes with an adjusted *P*-value <0.05 and that showed greater than a two-fold (up or down) change in expression were considered differentially expressed. As shown in Figure 4(a), a total of 34 DEGs (19 upregulated + 15 downregulated) were identified in the aprepitant/itraconazole treated sample (Figure 4(a) and Table 2). Genes involved in ergosterol synthesis (*ERG1, ERG2, ERG10, ERG24*), stress responses (*DDR48* and *RTA3*), and drug transporters (*CDR1, MDR1, DUR3*) were upregulated in the aprepitant/itraconazole treated sample. Additionally, the iron-regulated 1,3-beta glucan-linked cell wall gene (*PIR1*) and the vacuolar calcium P-type ATPase (*PMC1*) were also among the upregulated DEGs. On the other hand, the aprepitant/itraconazole combination resulted in the downregulation of genes responsible for metal ion transport such as *FTR1* (iron transporter), *ZTR2* (zinc transporter), and *CTR1*, which is important for both iron and copper transport. Also, several high-affinity glucose transporters (*HGT6, HGT8, HGT12*) were among the downregulated DEGs in the aprepitant/itraconazole treated sample.

**Table 2.** List of differentially expressed genes (DEGs) for *C. auris* AR0390 treated with a combination of aprepitant (10 µg/ml) and itraconazole (1 µg/ml)

ID	Name	Log_2_ Fold Change	*P-*value	CGD* Description
40027169	*HGT6*	−4.90	3.1 x 10^−20^	Putative high-affinity major facilitator superfamily (MFS) glucose transporter
40027170	*HGT8*	−3.29	5.5 x 10^−10^	High-affinity glucose transporter of the major facilitator superfamily
40029385	*LIP2*	−3.09	1.5 x 10^−6^	Secreted lipase, expressed in the alimentary tract, but not during oral infection
40027445	*FTR1*	−2.63	1.5 x 10^−6^	High-affinity iron permease, required for mouse virulence
40029696	-	−2.61	1.2 x 10^−3^	Protein of unknown function
40028877	*ZRT2*	−2.48	9.1 x 10^−6^	Zinc transporter, essential for zinc uptake
40027663	*CTR1*	−2.24	7.2 x 10^−5^	Ortholog(s) have copper/iron transmembrane transporter activity
40026070	*orf19.6983*	−2.21	8.5 x 10^−5^	Protein of unknown function, repressed by nitric oxide
40029380	*TYE7*	−2.20	1.7 x 10^−4^	Transcription factor, control of glycolysis, required for biofilm formation
40028922	*CDG1*	−2.04	5.2 x 10^−4^	Putative cysteine dioxygenases, role in conversion of cysteine to sulfite
40029539	*ADH1*	−2.03	5.2 x 10^−4^	Alcohol dehydrogenase
40026699	*PDC11*	−1.87	1.9 x 10^−3^	Pyruvate decarboxylase, antigenic fluconazole, farnesol induced
40026939	*HGT12*	−1.82	3.2 x 10^−3^	Glucose, fructose, mannose transporter, major facilitator superfamily
40029413	*JEN2*	−1.79	4.1 x 10^−3^	Dicarboxylic acid transporter, regulated by glucose repression
40027889	*YHB1*	−1.78	4.1 x 10^−3^	Nitric oxide dioxygenase, acts in nitric oxide scavenging/detoxification
40025324	*PMC1*	1.78	2.3 x 10^−5^	Vacuolar calcium P-type ATPase, mutants show increased resistance to fluconazole
40027146	*DUR3*	1.82	1.8 x 10^−5^	High-affinity spermidine transporter
40030172	*ERG2*	1.88	9.2 x 10^−6^	C-8 sterol isomerase, an enzyme important for ergosterol biosynthesis
40029187	*MDR1*	1.97	3.7 x 10^−6^	Plasma membrane MDR/MFS multidrug efflux pump
40026984	*orf19.3988*	1.98	5.8 x 10^−6^	Putative adhesin-like protein
40025409	*ERG1*	1.99	2.8 x 10^−6^	Squalene epoxidase, ergosterol biosynthesis
40030782	*ERG10*	2.04	5.9 x 10^−6^	Acetyl-CoA acetyltransferase, role in ergosterol biosynthesis
40025820	-	2.04	2.3 x 10^−6^	Protein of unknown function
40029256	*PIR1*	2.22	2.3 x 10^−7^	1,3-beta-glucan-linked cell wall protein, iron regulated
40029691	-	2.23	2.7 x 10^−7^	Protein of unknown function
40025314	*CDR1*	2.25	1.5 x 10^−7^	Multidrug transporter of ABC superfamily
40029715	*orf19.5169*	2.29	9.7 x 10^−7^	Has domain(s) with predicted amidase activity
40025414	-	2.34	6.6 x 10^−8^	Protein of unknown function
40027963	*INO1*	2.38	7.9 x 10^−8^	nositol-1-phosphate synthase/glycosylation predicted
40028953	*CAN1*	2.53	1.2 x 10^−8^	Basic amino acid permease
40028243	*ERG24*	2.60	2.2 x 10^−9^	C-14 sterol reductase has a role in ergosterol biosynthesis
40027842	*RCT1*	2.72	4.8 x 10^−10^	Fluconazole-induced protein, required for caspofungin tolerance
40029690	*RTA3*	3.02	1.4 x 10^−11^	Putative lipid translocase influences the susceptibility of *C. albicans* to fluconazole
40030199	*DDR48*	3.19	1.1 x 10^−12^	Immunogenic stress-associated protein

*Descriptions of the identified DEGs obtained from the *Candida* Genome Database (CGD) combined with a tBLASTx search.

With regards to single treatments with either aprepitant or itraconazole, no significant change in *C. auris’* transcriptome was observed in the aprepitant treated condition (Figure 4(c)). However, a total of 18 DEGs (10 upregulated + 8 downregulated) were detected in samples treated with itraconazole alone (Figure 4(d) and Table 3). Among the upregulated genes in the itraconazole treated condition were amino acid permeases (*DIP5* and *CAN1*), stress-associated genes (*DDR48* and *PGA31*), ergosterol biosynthesis (*ERG24*), and a redox related gene, ferric reductase (*FRE3*).

**Table 3.** List of differentially expressed genes (DEGs) for *C. auris* AR0390 treated with itraconazole (1 µg/ml)

ID	Name	Log_2_ Fold Change	*P-*value	CGD* Description
40027169	*HGT6*	−2.9273	9.6 x 10^−8^	Putative MFS glucose transporter
40027170	*HGT8*	−2.7557	6.1 x 10^−7^	High-affinity glucose transporter of the major facilitator superfamily
40029385	*LIP2*	−2.3233	4.5 x 10^−4^	Secreted lipase
40029539	*ADH1*	−2.3056	7.7 x 10^−5^	Alcohol dehydrogenase
40027889	*YHB1*	−2.245	1.2 x 10^−4^	Nitric oxide dioxygenase
40029413	*JEN2*	−2.1117	4.4 x 10^−4^	Dicarboxylic acid transporter
40028527	*TDH3*	−2.0678	4.8 x 10^−4^	NAD-linked glyceraldehyde-3-phosphate dehydrogenase
40030546	*DAL81*	−2.0262	3.7 x 10^−4^	RNA polymerase II repressing transcription factor binding activity
40025268	*PGA30*	1.9233	3.1 x 10^−4^	Predicted GPI-anchored protein
40026925	*DIP5*	1.9692	4.7 x 10^−4^	Dicarboxylic amino acid permease
40028209	*PRA1*	2.0941	4.5 x 10^−4^	Cell surface protein that sequesters zinc from host tissue
40027842	*RCT1*	2.1026	4.5 x 10^−4^	Fluconazole-induced protein, required for caspofungin tolerance
40029187	*MDR1*	2.1045	4.4 x 10^−4^	Plasma membrane MDR/MFS multidrug efflux pump
40030199	*DDR48*	2.1539	3.7 x 10^−4^	Stress-associated protein, induced by benomyl/caspofungin/ketoconazole
40030120	*PGA31*	2.3457	7.7 x 10^−5^	Protein associated with cellular response to chemical stimulus
40028243	*ERG24*	2.3585	5.2 x 10^−5^	C-14 sterol reductase has a role in ergosterol biosynthesis
40025718	*FRE3*	2.7106	1.09 x 10^−6^	Protein with similarity to ferric reductase Fre10p
40028953	*CAN1*	3.4505	3.5 x 10^−10^	Basic amino acid permease

* Descriptions of the identified DEGs obtained from the *Candida* Genome Database (CGD) combined with a tBLASTx search.

**Figure 5.**
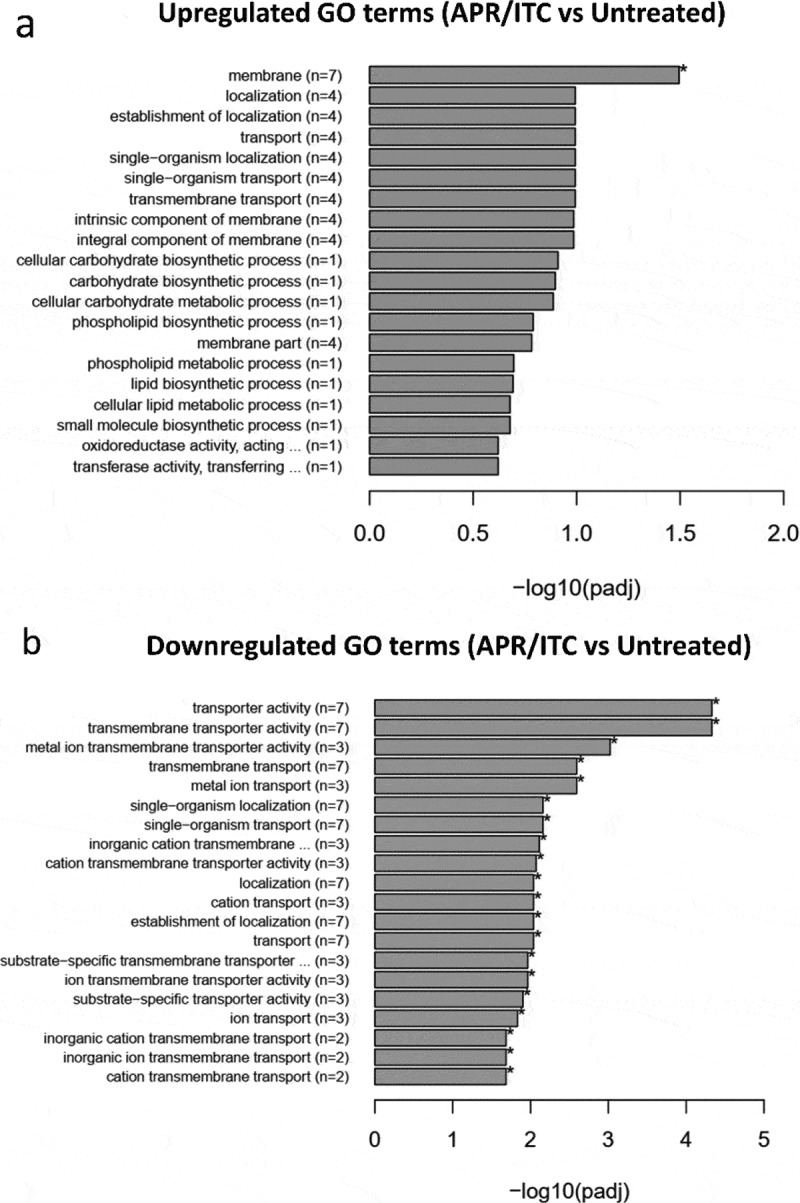
**Enrichment analysis of all GO (gene ontology) terms of DEGs identified from the aprepitant/itraconazole combination (APR/ITC) versus the untreated control**. GO analysis of DEGs was implemented by the ClusterProfiler R package and a *P*-value ≤ 0.05 was used as the cutoff parameter. Panel (a) displays up-regulated GO terms and panel (b) displays down-regulated DEGs

In order to obtain an overall insight into the impact of aprepitant/itraconazole on the *C. auris* transcriptome, we performed a Gene Ontology (GO) analysis of the identified DEGs. Twenty-six GO terms were significantly overrepresented (enriched) in the aprepitant/itraconazole treated condition. However, no significant overrepresented GO terms were found in the itraconazole treated condition. Among the enriched GO terms, only one GO term related to membrane components was upregulated in the aprepitant/itraconazole sample (Figure 5(a) and Supplementary Table S4). However, a total of 25 GO terms were found to be downregulated in the aprepitant/itraconazole treated sample (Figure 5(b) and Supplementary Table S5). Notably, 18 downregulated GO terms were found to be associated with membrane transport processes, of which 11 GO terms were involved in metal ions and cation transport, indicating the negative impact that aprepitant/itraconazole combination has on the ions homeostasis in *C. auris*. Of note, since all genes that demonstrated differential expression in response to aprepitant/itraconazole treatments have orthologs in other *Candida* species, we expect this combination would have a similar mechanism of action in all susceptible species.

### Validation of RNA-Seq data

**Figure 6.**
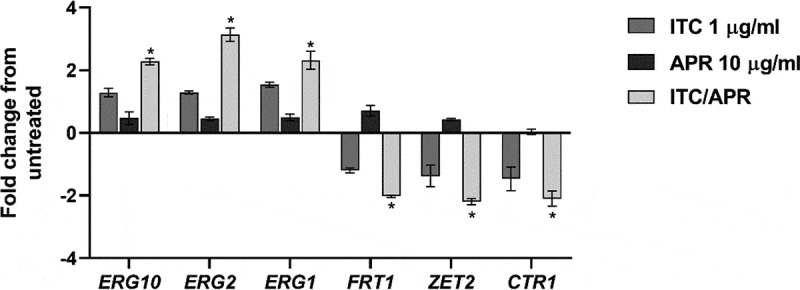
**Quantification of *C. auris* selected genes that were differentially expressed only in the aprepitant/itraconazole combination (APR/ITC)**. Log phase *C. auris* AR0390 cells were exposed to APR (10 µg/ml), ITC (1 µg/ml), or a combination of both drugs for three hours. Following treatment, cells were harvested, lysed, and RNA was extracted. Gene expression was determined by quantitative RT-PCR using *ACT1* as a housekeeping gene and the untreated sample as a reference control. An absolute two-fold change relative to the untreated control was set as a statistical cutoff value. The result is presented as mean ± SD

Based on the RNA-Seq data, we selected six DEGs (three downregulated and three upregulated) exhibiting a broad range of differential expression for validation by RT-qPCR. Of note, three genes selected for analysis are involved in ergosterol biosynthesis and three genes are involved in metal ion homeostasis. Consistent with the RNA-Seq data, the qPCR analysis showed significant differential expression of the tested genes and confirmed the significant overexpression of ergosterol biosynthesis-related genes (*ERG1, ERG2, ERG10*) and the significant downregulation of metal transport-related genes (*FTR1, ZRT2*, and *CTR1*) only in the cells treated with the aprepitant/itraconazole combination (Figure 6).

### Effect of aprepitant on ROS production

**Figure 7.**
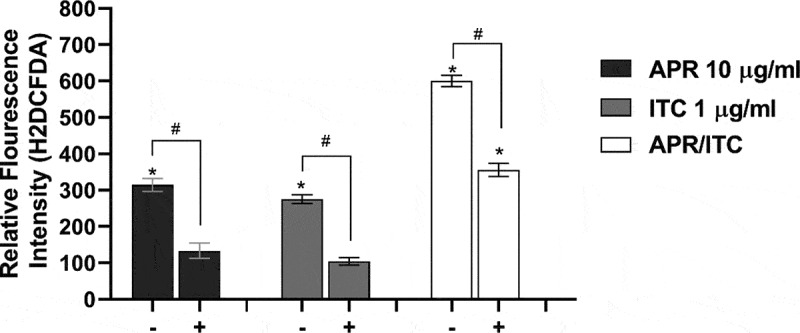
**Effect of aprepitant/itraconazole on the ROS levels in the presence (+) or absence (-) of iron**. Carboxy-H2DCFDA probe was used to analyze ROS levels within *C. auris* AR0390 in the presence (+) or absence of iron source (FeSO4 50 µM). PBS suspensions of *C. auris* cells, at 1× 10^6^ CFU/ml, were treated with either aprepitant (APR, 10 µg/ml), itraconazole (ITC, 1 µg/ml), or a combination of both for 3 h, and then were treated with H2DCFDA for 1 h, before measuring the fluorescence intensity as an indication of ROS levels. Data are shown as means ± SD. An asterisk (*) indicates statistical significance (*P* < 0.05) between treated and untreated groups (*P* < 0.05), whereas #, indicates statistical significance (*P* < 0.05) between treatment groups in the presence or absence of iron. Statistical significance was assessed by one-way ANOVA using Dunnett’s test for multiple comparisons

Since metal ion homeostasis is critical for multiple biological processes, including redox regulatory mechanisms, we were curious to examine the effect of the aprepitant/itraconazole combination on the ability of *C. auris* to detoxify reactive oxygen species (ROS). As presented in Figure 7, aprepitant (at 10 µg/ml) significantly increased the intracellular ROS levels by ~ 300% relative to the untreated control, while itraconazole treatment (at 1 µg/ml) significantly increased the ROS levels by 270% relative to the untreated control. On the other hand, the aprepitant/itraconazole combination resulted in a more prominent effect by increasing the ROS production by ~ 600% relative to the untreated control. Interestingly, when the assay medium was supplemented with iron, we noticed that *C. auris* cells generated significantly lower levels of ROS as compared to iron-deficient media (Figure 7). This result indicates that both aprepitant and the aprepitant/itraconazole combination can interfere significantly with ROS detoxification in *C. auris*, apparently through the interference with metal ion transport.

### Effect of metal ion supplementation on the synergistic relationship between aprepitant and itraconazole

**Figure 8.**
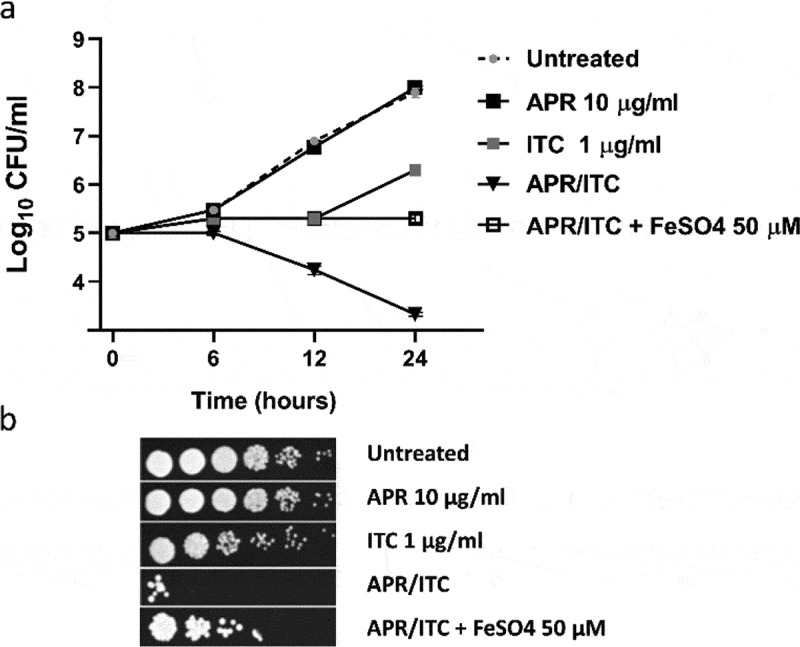
Effect of iron supplementation on the antifungal activity of aprepitant/itraconazole (APR/ITC). (a) Effect of iron supplement (FeSO_4_, at 50 µM) on the time-kill kinetics of aprepitant (10 µg/ml), itraconazole (1 µg/ml), or a combination of both drugs against *C. auris* AR0390. (b) Spot assays demonstrated the ability of iron supplementation to reverse the antifungal effect exerted by the aprepitant/itraconazole combination (10/1 µg/ml)

Based on the data obtained from the transcriptomic analysis, several genes involved in metal ion transport were found to be downregulated in cells treated with the aprepitant/itraconazole combination. Thus, it was rational to investigate whether supplementation with exogenous metal ions would reverse the synergistic interaction observed between aprepitant and itraconazole. As shown in Figure 8(a), iron supplementation interfered significantly with the synergistic relationship of aprepitant and itraconazole, as demonstrated by a time-kill assay. Ferrous sulfate (at 50 µM) completely negated the fungicidal activity of the aprepitant/itraconazole combination against the test isolate (*C. auris* AR0390). This effect was further demonstrated by a spotting assay where iron supplementation restored the visible growth of *C. auris* colonies on YPD agar and eliminated the fungicidal effect of the aprepitant/itraconazole combination (Figure 8(b)). It should be noted that copper supplementation did not affect the antifungal activity of aprepitant/itraconazole (data not shown). Also, it was not possible to examine the effect of zinc supplementation on the relationship between aprepitant and itraconazole, as we noticed an immediate precipitation reaction when zinc sulfate was mixed with aprepitant. However, these results indicate that interference with iron homeostasis is a key mechanism that contributes to the synergistic relationship observed between aprepitant and itraconazole against *C. auris*.

### Efficacy of aprepitant/itraconazole in a Caenorhabditis elegans model of C. auris infection

**Figure 9.**
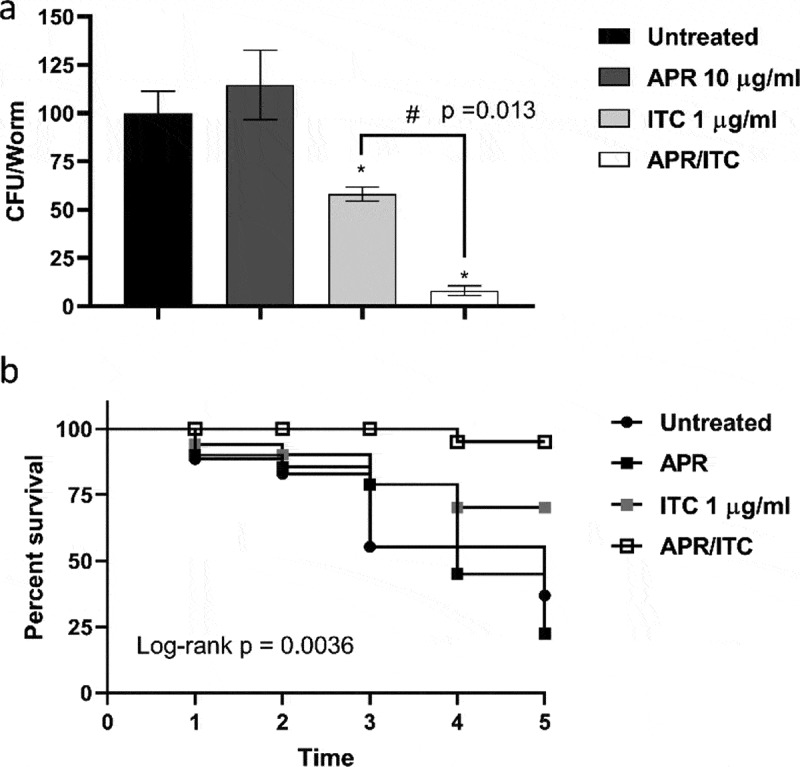
***In vivo* efficacy of aprepitant/itraconazole in *Caenorhabditis elegans* infected with *C. auris***. *C. elegans* nematodes were infected with 1 × 10^7^ CFU *C. auris* AR0390 and then treated with aprepitant (APR), at 10 µg/ml, and itraconazole (ITC), at 1 µg/ml, either alone or in combination. Untreated worms served as a negative control. (a) *C. auris* AR0390 CFU burden/worm 24 h post-treatment. An asterisk (*) indicates statistical significance (*P* < 0.05) relative to the untreated control, while # indicates statistical significance (*P* < 0.05) compared to the ITC treatment. Statistical significance was assessed by one-way ANOVA using Dunnett’s test for multiple comparisons. (b) Kaplan-Meier survival curve, assessed by log-rank test for significance, of *C. elegans* nematodes infected by *C. auris* AR0390 and treated with APR (10 µg/ml), ITC (1 µg/ml), or a combination of both agents

To further corroborate the *in vitro* synergistic relationship observed between aprepitant and itraconazole, we tested the *in vivo* efficacy of aprepitant/itraconazole using *C. elegans* as an infection model. As shown in Figure 9(a), treatment with aprepitant alone at 10 µg/ml failed to reduce the fungal CFU burden in *C. elegans* nematodes infected with *C. auris* AR0390. However, itraconazole at 1 µg/ml (1 × MIC) was able to significantly reduce the fungal CFU burden by ~42 ± 3.6%, compared to the untreated control. Interestingly, the aprepitant/itraconazole combination was able to significantly reduce the *C. auris* CFU burden in the infected nematodes by ~92 ± 2.4%, compared to the untreated control.

Next, we assessed the effect of aprepitant/itraconazole on the survival of infected nematodes. As shown in Figure 9(b), only 20% of the untreated *C. elegans* nematodes survived *C. auris* infection for five days post-infection. As expected, aprepitant at 10 µg/ml did not improve the survival of infected nematodes with respect to the untreated control. However, itraconazole at 1 µg/ml (1 × MIC) enhanced the survival of the infected worms by ~ 40% (*P* < 0.05), compared to only 20% in the untreated group. On the other hand, the aprepitant/itraconazole combination significantly enhanced the survival of the infected nematodes as ~ 90% (*P* < 0.05) of *C. elegans* remained alive for five days. Altogether, these findings indicate that aprepitant/itraconazole has a potent *in vivo* efficacy of treating *C. auris* infection of *C. elegans* and warrants further investigation in higher animal models.

## Discussion

Drug repurposing is a valuable tool to identify novel antimicrobial agents and co-drugs capable of enhancing the activity of existing antimicrobials [27,43–46]. The risk posed by the recently identified fungal pathogen *C. auris* has prompted CDC to classify *C. auris* as an urgent threat that requires immediate action [3]. A hallmark trait of *C. auris* is its ability to resist multiple antifungal therapeutics, especially azoles. Here, we utilized a whole-cell screening assay to evaluate the azole chemosensitizing activity of ~1547 FDA-approved drugs and clinical molecules against the multidrug-resistant *C. auris* isolate AR0390. The screening data (unpublished) revealed aprepitant, an NK1 antagonist used for the treatment of chemotherapy-associated emesis, was able to restore the antifungal activity of fluconazole against the test isolate. This observation encouraged the exploration of the azole chemosensitizing activity of aprepitant against a panel of ten *C. auris* isolates, in addition to several clinically important *Candida* species. Checkerboard data indicate that aprepitant enhances the antifungal activity of azole drugs against various *Candida* species. More specifically, aprepitant interacted synergistically with itraconazole against the majority of *Candida* isolates tested. Additionally, combining aprepitant with itraconazole changed the fungistatic nature of itraconazole and produced a prominent fungicidal effect against *C. auris* AR0390 as demonstrated by the time-kill study. From a clinical point of view, this observation is of interest since fungicidal therapeutics are expected to improve the clinical outcome of invasive *Candida* infections, especially in patients with compromised immune systems [47,48].

A well-documented virulence factor of *Candida* species is the notable ability to form adherent structures, known as biofilms, on surfaces of medical devices [49–51]. Given the potent synergistic interaction and the fungicidal effect displayed by the aprepitant/itraconazole combination against planktonic *C. auris* cells, it was of interest to examine whether a similar effect could exist against the difficult-to-treat fungal biofilms. While neither aprepitant nor itraconazole alone was able to interfere with the biofilm-forming ability of *C. auris*, the aprepitant/itraconazole combination was effective at reducing the metabolic activity of fungal cells residing within premature and mature biofilms. These observations may be of clinical importance since biofilms are known to hinder the penetration of antifungal drugs and host immune effectors and also trigger the formation of *Candida* persister cells, leading to poor clinical outcomes [52,53].

To gain insight into the molecular mechanism by which aprepitant interacts synergistically with azole drugs against *C. auris*, we performed comparative transcriptomic profiling of *C. auris* cells treated with DMSO (1%), aprepitant (10 µg/ml), itraconazole (1 µg/ml), or the aprepitant/itraconazole combination (10/1 µg/ml). Our data indicate that aprepitant, at the tested concentration, has no significant effect on *C. auris’* transcriptome. However, a total of 18 and 34 genes displayed significant changes in expression in groups treated with itraconazole and aprepitant/itraconazole respectively, compared to the untreated control. We focused our attention on the differentially expressed genes that were found to be specific to the aprepitant/itraconazole treated group. We noticed that several genes involved in metal ions transport were significantly downregulated in cells treated with aprepitant/itraconazole, relative to the untreated control. This observation suggests the aprepitant/itraconazole combination can interfere with metal ion homeostasis in *C. auris*. Since metal ions are key components of several essential genes, it is expected that disturbances in metal ion homeostasis would compromise multiple vital biological processes. One striking example is the critical role of metal ions, particularly iron, in the activity of many enzymes involved in the detoxification of reactive oxygen species (ROS) [54–56]. Thus, we sought to examine the effect of aprepitant/itraconazole on the ROS detoxification ability of *C. auris*. Interestingly, aprepitant by itself compromised the ROS detoxification ability of *C. auris*, resulting in elevated ROS levels (eight-times higher compared to the untreated control). This effect was even more pronounced with the aprepitant/itraconazole combination which generated ~ 15-times more ROS levels, compared to the untreated control. These increases in ROS levels could explain the observed fungicidal activity against planktonic and biofilms of *C. auris* as previously demonstrated in other studies [57–59]. Further supporting the critical role of iron homeostasis in controlling the intracellular ROS levels, we noticed that in iron supplemented media, *C. auris* cells treated with aprepitant/itraconazole produced significantly lower ROS levels, compared to iron-depleted media. Interestingly, similar observations have been recorded in plant and mammalian cells whereas high ROS levels were produced in iron depleting conditions [60,61], indicating that iron homeostasis has a conservative protective role against the ROS formation.

In *Saccharomyces cerevisiae*, iron homeostasis is known to be an important regulator of ergosterol biosynthesis [62]. In iron depleting conditions, yeast cells were reported to express higher mRNA levels of several *ERG* genes such as *ERG1, ERG7*, and *ERG11* [62]. Consistent with this phenomenon, we noticed that several genes involved in ergosterol biosynthesis were significantly upregulated such as *ERG1, ERG2*, and *ERG10*. Our data also shows increased mRNA levels of *ERG11*, however, this upregulation didn’t meet our strict criteria for statistical significance. This upregulation of ERG genes could be viewed as a compensatory mechanism by which *C. auris* cells try to adapt to the reduced ergosterol content. Indeed, previous studies have shown that ergosterol depletion causes the upregulation of several genes involved in ergosterol biosynthesis [63,64].

Notably, iron homeostasis has been regarded as a potential target for other azole chemosensitizing agents such as doxycycline and lactoferrin [65,66]. Disturbance in iron transport was reported to affect the membrane permeability and enhance the activity of drugs that target cell membrane biosynthetic pathways such as azoles [67]. Additionally, iron depletion is thought to suppress the calcineurin pathway, leading to impairment of drug-induced stress responses in *Candida* [68]. Thus, we were interested to examine whether iron supplementation would weaken the synergistic interaction observed between aprepitant and itraconazole against *Candida*. Indeed, iron supplementation negated the fungicidal activity of aprepitant/itraconazole as shown in our time-kill kinetics. However, even in the presence of iron, the aprepitant/itraconazole combination was still able to exert a noticeable fungistatic activity against *C. auris*. This suggests that other additional mechanisms may contribute to the synergistic relationship between aprepitant and itraconazole. Of note, in contrast to iron, copper supplementation did not interfere with the antifungal activity of the aprepitant/itraconazole combination.

To conclude, our data indicate that aprepitant was able to enhance the antifungal activity of azole drugs, particularly itraconazole. The aprepitant/itraconazole combination displayed broad-spectrum antifungal activity and was fungicidal against planktonic and biofilms of *C. auris*, suggesting a promising clinical indication for the treatment of azole-recalcitrant infections. Furthermore, this novel drug combination demonstrated potent *in vivo* efficacy in a *C. elegans* infection model. The synergistic relationship between aprepitant and itraconazole appears to be mediated through potent interference with metal ion homeostasis, and the subsequent compromise in ROS detoxifying mechanisms and ergosterol biosynthesis, however, further molecular studies are needed to fully understand the mechanism of the azole chemosensitizing activity of aprepitant and its effect on the ergosterol pathway. Moreover, efficacy in higher animal models and further clinical studies are needed to fully assess the potential clinical use of aprepitant/itraconazole as a novel drug combination with promising antifungal activity against emergent multidrug-resistant *C. auris*.

## Supplementary Material

Supplemental Material
