# Supplementary material for: Aprepitant, an antiemetic agent, interferes with metal ion homeostasis of Candida auris and displays potent synergistic interactions with azole drugs
Source: Virulence. 2020 Oct 26;11(1):1466–81. doi: 10.1080/21505594.2020.1838741 (PMC7588212; doi:10.1080/21505594.2020.1838741)
Supplement: Supplemental Material [file KVIR_A_1838741_SM7178.docx]

**Aprepitant, an antiemetic agent, interferes with metal ion homeostasis of *Candida auris* and displays potent synergistic interactions with azole drugs**

Hassan E. Eldesouky^1,2^, Nadia A Lanman ^1,3^_,_ Tony R. Hazbun ^4^, and Mohamed N. Seleem ^1,2*^

^1^Department of Comparative Pathobiology, College of Veterinary Medicine, Purdue University, West Lafayette, IN 47907, USA

^2^Department of Biomedical Sciences and Pathobiology, Virginia-Maryland College of Veterinary Medicine, Virginia Polytechnic Institute and State University, Blacksburg, VA, 24061, USA.

^3^Purdue University Center for Cancer Research, Purdue University, West Lafayette, IN 47907, USA

^4^Department of Medicinal Chemistry and Molecular Pharmacology, College of Pharmacy, Purdue University, West Lafayette, Indiana 47907, USA

*Correspondence:

seleem@vt.edu

**Supplementary Table S1**. List of strains used in this study

| **Fungal Strains** | **Source** | **Description** |
| --- | --- | --- |
| *C. auris 381* | CDC | Drug sensitive isolate |
| *C. auris 382* | CDC | Drug sensitive isolate |
| *C. auris 383* | CDC | Clinical isolate exhibiting resistance to fluconazole |
| *C. auris 384* | CDC | Clinical isolate exhibiting resistance to fluconazole and Voriconazole |
| *C. auris* 385 | CDC | Resistant to fluconazole, itraconazole, voriconazole, and amphotericin B |
| *C. auris* 386 | CDC | Resistant to fluconazole, voriconazole, and amphotericin B |
| *C. auris 387* | CDC | Drug sensitive isolate |
| *C. auris* 388 | CDC | Resistant to fluconazole, itraconazole, voriconazole, and amphotericin B- overexpression of *CDR1* |
| *C. auris* 389 | CDC | Resistant to fluconazole, itraconazole, voriconazole, and amphotericin B- overexpression of *CDR1* |
| *C. auris* 390 | CDC | Resistant to fluconazole, itraconazole, and amphotericin B- overexpression of *MDR1* and *CDR1* |
| *C. albicans* SC5314 | ATCC | Wild-type strain |
| *C. albicans ATCC MYA-573* | ATCC | Fluconazole-resistant bloodstream isolate (from an AIDS patient in Germany) |
| *C. albicans* TWO7241 | Professor Theodore White (University of Missouri-Kansas City) | Fluconazole-resistant clinical isolate that has increased efflux activity (*MDR1* overexpression) and overexpression of the azole target (*ERG11*) |
| *C. albicans* TWO7243 |  | Fluconazole- and itraconazole-resistant clinical isolate that has increased mRNA levels of *CDR1*, *MDR1*, and *ERG11* |
| *C. glabrata* ATCC-66032 | ATCC | Not available |
| *C. glabrata* ATCC-64677 | ATCC | Not available |
| *C. glabrata* ATCC-15126 | ATCC | Not available |
| *C. krusei* CAB39-6420 | Bei Resources | Human blood isolate |
| *C. krusei* ATCC-14243 | ATCC | Not available |
| *C. krusei* ATCC-34135 | ATCC | Clinical specimen isolated in Minnesota, USA |
| *C. tropicalis* ATCC-13803 | ATCC | Not available |
| *C. tropicalis* ATCC-1369 | ATCC | Not available |
| *C. parapsilosis* ATCC-22019 | ATCC | Clinical specimen isolated in Puerto Rico |
| *C. parapsilosis* CAB50-2638 | Bei Resources | Human blood isolate |

**Supplementary Table S2**. List of primers

| Gene name | Primer | |
| --- | --- | --- |
| *ACT1* | Forward | GAAGGAGATCACTGCTTTAGCC |
|  | Reverse | GAGCCACCAATCCACACAG |
| *FTR1* | Forward | GTCAACTGCTGCAATCCTGA |
|  | Reverse | TTCTTGCAGAATGGCAAGTG |
| *CTR1* | Forward | TACTATGGATCACGCCCACA |
|  | Reverse | CACATGTTCATGCCCATCAT |
| *ZTR2* | Forward | TGTTGGTCAGGTTGTGCAAT |
|  | Reverse | AGAAACACCAACCAGGCAAC |
| *ERG2* | Forward | CTTTGACGACTGGGTGTTCA |
|  | Reverse | CCACAGAAGTGCCGAAAAA |
| *ERG10* | Forward | CTCGGAAACCAACACCAAAG |
|  | Reverse | TCAAGGGCACCAGAGGTAAG |
| *ERG1* | Forward | CCGTGCTCATTTACCAGACA |
|  | Reverse | AGGGCAACACCTCATCTCTC |

**Supplementary Table S3**. Effect of aprepitant on the antifungal activity of itraconazole (ITC) against clinically relevant *Candida* species.

| **Isolates** | **MIC (µg/mL)** | | | | **ΣFICI*** | **Interaction** |
| --- | --- | --- | --- | --- | --- | --- |
|  | **Aprepitant** | | **ITC** | |  |  |
|  | **Alone** | **Combined** | **Alone** | **Combined** |  |  |
| *C. albicans* SC-5314 | > 128 | 8 | 0.25 | 0.0156 | 0.13 | SYN |
| *C. albicans* ATCC-MYA 573 | > 128 | 8 | 1 | 0.25 | 0.31 | SYN |
| *C. albicans* TWO743 | > 128 | 8 | 1 | 0.25 | 0.31 | SYN |
| *C. albicans* TWO741 | > 128 | 8 | 0.5 | 0.0625 | 0.19 | SYN |
| *C. glabrata* ATCC-64677 | > 128 | 2 | 0.5 | 0.5 | 1.02 | IND |
| *C. glabrata* ATCC-15126 | > 128 | 2 | 0.25 | 0.25 | 1.02 | IND |
| *C. glabrata* ATCC-66032 | > 128 | 2 | 0.5 | 0.5 | 1.02 | IND |
| *C. krusei* CAB 396420 | > 128 | 8 | 0.5 | 0.0625 | 0.187 | SYN |
| *C. krusei* ATCC-14243 | > 128 | 8 | 0.25 | 0.015 | 0.13 | SYN |
| *C. krusei* ATCC-34135 | > 128 | 8 | 0.5 | 0.062 | 0.19 | SYN |
| *C. tropicalis* ATCC-13803 | > 128 | 8 | 0.25 | 0.062 | 0.31 | SYN |
| *C. tropicalis* ATCC-1369 | > 128 | 2 | 0.5 | 0.031 | 0.08 | SYN |
| *C. parapsilosis* ATCC-22019 | > 128 | 8 | 0.25 | 0.062 | 0.31 | SYN |
| *C. parapsilosis* CAB-502638 | > 128 | 8 | 0.25 | 0.062 | 0.31 | SYN |

^*^ΣFICI (fractional inhibitory concentration index) is used to measure the interaction between the tested combinations. ΣFICI interpretation corresponded to the following definitions: synergism (SYN), ΣFICI ≤ 0.5; additivity (ADD), ΣFICI > 0.5 and ≤ 1; and indifference (IND), ΣFICI > 1 and ≤ 4.

**Supplementary Table S4** - Significantly upregulated GO terms in APR/ITC treatment group

| **Category** | **GOID** | **Description** | **Gene**  **Ratio** | **BgRatio** | **P value** | **Padj** | **Gene**  **ID** | **Count** |
| --- | --- | --- | --- | --- | --- | --- | --- | --- |
| CC | GO:0016020 | membrane | 7/7 | 466/926 | 0.00799 | 0.03197 | 40025314/40025409/40027146/40028243/40028953/40029187/40029690 | 7 |

**Supplementary Table S5-** Significantly downregulated GO terms in APR/ITC treatment group

| **Category** | **GOID** | **Description** | **Gene**  **Ratio** | **BgRatio** | **P value** | **Padj** | **Gene ID** | **Count** |
| --- | --- | --- | --- | --- | --- | --- | --- | --- |
| BP | GO:0030001 | Metal ion transport | 3/11 | 22/1815 | 0.00024 | 0.00255 | 40027445/40027663/40028877 | 3 |
| BP | GO:0055085 | Transmembrane transport | 7/11 | 267/1815 | 0.00027 | 0.00255 | 40026939/40027169/40027170/40027445/40027663/40028877/40029413 | 7 |
| BP | GO:0044765 | Single-organism transport | 7/11 | 341/1815 | 0.00128 | 0.00693 | 40026939/40027169/40027170/40027445/40027663/40028877/40029413 | 7 |
| BP | GO:1902578 | Single-organism localization | 7/11 | 348/1815 | 0.00146 | 0.00693 | 40026939/40027169/40027170/40027445/40027663/40028877/40029413 | 7 |
| BP | GO:0006810 | Transport | 7/11 | 397/1815 | 0.0033 | 0.00912 | 40026939/40027169/40027170/40027445/40027663/40028877/40029413 | 7 |
| BP | GO:0051234 | Establishment of localization | 7/11 | 400/1815 | 0.00346 | 0.00912 | 40026939/40027169/40027170/40027445/40027663/40028877/40029413 | 7 |
| BP | GO:0006812 | Cation transport | 3/11 | 55/1815 | 0.00366 | 0.00912 | 40027445/40027663/40028877 | 3 |
| BP | GO:0051179 | Localization | 7/11 | 407/1815 | 0.00384 | 0.00912 | 40026939/40027169/40027170/40027445/40027663/40028877/40029413 | 7 |
| BP | GO:0006811 | Ion transport | 3/11 | 69/1815 | 0.00697 | 0.01472 | 40027445/40027663/40028877 | 3 |
| BP | GO:0098655 | Cation transmembrane transport | 2/11 | 30/1815 | 0.01325 | 0.02063 | 40027445/40027663 | 2 |
| BP | GO:0098660 | Inorganic ion transmembrane transport | 2/11 | 30/1815 | 0.01325 | 0.02063 | 40027445/40027663 | 2 |
| BP | GO:0098662 | Inorganic cation transmembrane transport | 2/11 | 30/1815 | 0.01325 | 0.02063 | 40027445/40027663 | 2 |
| BP | GO:0034220 | Ion transmembrane transport | 2/11 | 31/1815 | 0.01411 | 0.02063 | 40027445/40027663 | 2 |
| CC | GO:0044425 | Membrane part | 6/7 | 304/926 | 0.00612 | 0.03265 | 40026939/40027169/40027170/40027445/40027663/40029413 | 6 |
| CC | GO:0016020 | Membrane (n=7) | 7/7 | 466/926 | 0.00799 | 0.03265 | 40026939/40027169/40027170/40027445/40027663/40028877/40029413 | 7 |
| CC | GO:0016021 | Integral component of membrane | 5/7 | 239/926 | 0.01451 | 0.03265 | 40026939/40027169/40027170/40027663/40029413 | 5 |
| CC | GO:0031224 | Intrinsic component of membrane | 5/7 | 239/926 | 0.01451 | 0.03265 | 40026939/40027169/40027170/40027663/40029413 | 5 |
| MF | GO:0022857 | Transmembrane transporter activity | 7/12 | 153/2359 | 2.54E-06 | 4.68E-05 | 40026939/40027169/40027170/40027445/40027663/40028877/40029413 | 7 |
| MF | GO:0005215 | Transporter activity | 7/12 | 161/2359 | 3.60E-06 | 4.68E-05 | 40026939/40027169/40027170/40027445/40027663/40028877/40029413 | 7 |
| MF | GO:0046873 | Metal ion transmembrane transporter activity | 3/12 | 20/2359 | 0.00011 | 0.00095 | 40027445/40027663/40028877 | 3 |
| MF | GO:0022890 | Inorganic cation transmembrane transporter activity | 3/12 | 44/2359 | 0.00119 | 0.0077 | 40027445/40027663/40028877 | 3 |
| MF | GO:0008324 | Cation transmembrane transporter activity | 3/12 | 49/2359 | 0.00162 | 0.00845 | 40027445/40027663/40028877 | 3 |
| MF | GO:0015075 | Ion transmembrane transporter activity | 3/12 | 58/2359 | 0.00265 | 0.01086 | 40027445/40027663/40028877 | 3 |
| MF | GO:0022891 | Substrate-specific transmembrane transporter activity | 3/12 | 60/2359 | 0.00292 | 0.01086 | 40027445/40027663/40028877 | 3 |
| MF | GO:0022892 | Substrate-specific transporter activity | 3/12 | 66/2359 | 0.00384 | 0.01249 | 40027445/40027663/40028877 | 3 |
